# Prevalence and predictors of school truancy among adolescents in Sierra Leone: evidence from the 2017 Global School-based Health Survey

**DOI:** 10.1186/s12888-024-05888-9

**Published:** 2024-06-18

**Authors:** Augustus Osborne, Carol Labor, Camilla Bangura, Jia B. Kangbai

**Affiliations:** 1 Department of Biological Sciences, School of Basic Sciences, Njala University, PMB, Freetown, Sierra Leone; 2 Ministry of Health and Sanitation, Freetown, Sierra Leone; 3 Department of Environmental Health, School of Community Health Sciences, Njala University, PMB, Freetown, Sierra Leone

**Keywords:** Truancy, Adolescents, School, Sierra Leone

## Abstract

**Background:**

School truancy, deliberately skipping school without permission, is a complex issue with far-reaching consequences for individual students, education systems, and entire communities. While this phenomenon is not unique to Sierra Leone, the specific context of the post-conflict nation raises concerns about its potential impact on the country’s fragile rebuilding process. This study examines the prevalence and predictors of school truancy among adolescents in Sierra Leone.

**Methods:**

The study analysed the cross-sectional 2017 Global School-based Health Survey (GSHS) data in Sierra Leone, a nationally representative survey conducted among adolescents aged 10–19 years using a multistage sampling methodology. A weighted sample of 2,769 adolescents in Sierra Leone was included in the study. A multivariable binary regression analysis was used to examine the predictors of school truancy among adolescents. The regression results were presented using an adjusted odds ratio (AOR) with 95% confidence intervals (CI).

**Results:**

The prevalence of school truancy was 35% among adolescents in Sierra Leone. Adolescents who use alcohol (AOR = 2.28, 95% CI = 1.45, 3.58) and who have ever had sexual intercourse (AOR = 1.67, 95% CI = 1.10, 2.53) had higher odds of being associated with school truancy. Adolescents who planned suicide (AOR = 0.58, 95% CI = 0.36, 0.93) and whose parents did not intrude on their privacy (AOR = 0.66, 95% CI = 0.45, 0.97) had lower odds of being associated with school truancy.

**Conclusion:**

School truancy is a critical issue in Sierra Leone, demanding multi-pronged interventions at policy and practice levels. Addressing underlying causes like alcohol use, sexual behaviour, planned suicide, and parent’s intrusion of privacy is crucial. Key strategies include fostering positive school environments, providing mental health support, and improving parent-child communication.

## Introduction

School truancy is a severe problem affecting adolescents’ educational outcomes and well-being [1]. School truancy is skipping classes without purpose, not showing up at class and not attending school at all [1]. Previous studies among adolescents have reported that high-income countries have a lower truancy prevalence than low- and middle-income countries [2, 3], ranging from 59% in Zambia [2], 36.6% in Mozambique [4], 31% in Ghana [5], 30.8% in Malaysia [6], 25.7% in Tanzania [3], and 22% in Swaziland [7] to 12.0% found in the USA [8]. School truancy is a complex global issue with varying prevalence rates across countries. While obtaining a definitive worldwide truancy rate can be challenging due to differences in data collection methods and definitions, we explored the situation in Sierra Leone as comprehensive data on its prevalence in Sierra Leone remains scarce.

Truancy carries significant consequences for individuals and society [9, 10]. For adolescents, it is associated with lower academic achievement [7, 11, 12], increased risk of dropping out [11, 12], and reduced future employment opportunities [11]. Truants are also more vulnerable to risky behaviours like substance abuse and delinquent activities [12, 13]. On a broader scale, high truancy rates can impede educational development goals, weaken social cohesion, and hinder economic progress [11, 12].

Previous studies have reported that some of the factors that may contribute to truancy in school include socio-demographic characteristics such as age [4, 14, 15], gender [6, 7, 14, 15], grade level [7], hunger [3–5, 15], economic opportunities [9, 11], psychosocial factors such as alcohol use [5–7, 13, 15], smoking [4–6, 13, 15], bullying [4, 6, 7, 14, 15], drug use [6], loneliness [3, 4] and protective social factors such as peer support [4, 14, 15], teacher cooperation [10], and parental support [4–6, 8, 9, 14, 15]. These factors may interact and create a vicious cycle of truancy and poor outcomes.

The prevalence and correlates of school truancy among adolescents in Sierra Leone are poorly documented. Understanding the prevalence and correlates of truancy in Sierra Leone is a crucial first step in tackling this complex issue. Identifying risk factors associated with truancy can create positive and inclusive learning environments, prioritising student well-being and success. Therefore, this study aims to examine the prevalence and predictors of school truancy among adolescents in Sierra Leone and to identify the most effective interventions to prevent and reduce truancy using the first and only available Global School-based Health Survey data in Sierra Leone.

## Methods

### Data source and design

The study utilised a publicly accessible nationally representative cross-sectional dataset from the 2017 Sierra Leone Global School-based Student Health Survey (GSHS) [16]. The cross-sectional study employed a two-stage cluster sampling methodology to obtain a representative dataset encompassing all students aged ten to nineteen in grades Junior secondary school (JSS) 2, JSS 3, Senior secondary school (SSS) 2, and SSS 3. During the initial phase, schools were chosen based on a probability proportional to their enrollment size. For the second step, a random selection process was employed to choose classes; all students within the selected classes were considered eligible for selection. The response rates for the school, student, and overall populations were 94%, 87%, and 82%, respectively. The GSHS involved 2,798 student participants. The detailed GSHS description can be retrieved via https://extranet.who.int/ncdsmicrodata/index.php/catalog/772/study-description [17]. A weighted sample of 2,769 adolescents aged 10–19 years was included in our final analysis. The present study aligns with the Strengthening Reporting of Observational Studies in Epidemiology (STROBE) [18].

### Measures

The dependent variable was “school truancy” among adolescents, which was operationally defined as: How many days did you miss classes or school without permission in the past 30 days? The responses were: 0 days, 1 − 2 days, 3 − 5 days, 6 − 9 days, and ten or more. It was dichotomously recoded “1” if the respondent missed school and “0” if the respondent did not miss school without permission.

Based on the findings from similar studies [3–5, 14, 15], we selected several explanatory variables, such as gender, age, grade, marijuana use, suicidal ideation, suicidal planning and attempts, experiences of bullying, parental monitoring, engagement in sexual intercourse, parental understanding, parental bonding, parental intrusion of privacy, feelings of loneliness, and experiences of anxiety. The explanatory variables utilised in the analysis were designated and coded in the following manner: age (1 = ≤ 14, 0 = ≥ 15), sex (1 = male, 0 = female), grade (1 = 1–3, 0 = 4–5), truancy (1 = yes, 0 = no), and marijuana use (1 = yes, 0 = no). Furthermore, the psychosocial factors were categorised and coded as follows: suicidal ideation (1 = yes, 0 = no), planned suicide (1 = yes, 0 = no), attempted suicide (1 = yes, 0 = no), felt lonely (1 = yes, 0 = no), ever had sexual intercourse (1 = yes, 0 = no), and experience of bullying (1 = yes, 0 = no). Others were peer support (1 = yes, 0 = no), parental monitoring (1 = yes, 0 = no), parental bonding (1 = yes, 0 = no), parental intrusion of privacy (1 = yes, 0 = no), and parental understanding (1 = yes, 0 = no) See (Table 1).

### Data analysis

The data analysis was performed using SPSS software version 28.0. We used the complex sampling command on SPSS for weighting and complex sampling design. Percentages and frequencies were utilised to depict the prevalence and distribution of school truancy among adolescents in Sierra Leone. Subsequently, a Pearson chi-square test of independence was employed to investigate the factors that showed a significant connection with school truancy. Subsequently, a multivariable binary logistic regression analysis was employed to investigate the variables linked to school truancy among adolescents in Sierra Leone. The findings were reported using an adjusted odds ratio (aOR) and a 95% confidence interval (CI). The statistical significance level was established at *p* < 0.05 for the chi-square and regression analyses. We assess multicollinearity among independent variables using the variance inflation factor, and the results showed that the minimum and maximum VIFs were 1.02 and 1.63, respectively. The missing data were addressed through listwise deletion.

### Ethical consideration

Given that our study is based on examining a publicly available de-identified secondary dataset, there was no need for formal ethical approval to conduct this study. However, the World Health Organization (WHO) received ethical approval for the GSHS. In Sierra Leone, ethical clearance was sought from the Ministry of Health and Sanitation Ethics Board. Furthermore, child, parental or guardian consent forms were obtained from adolescent participants under 18 years before their inclusion in the survey. Additionally, the WHO ensured respondents 18 years old and above provided verbal and written informed consent.

## Results

### Distribution of school truancy among adolescents in Sierra Leone

Table 1 shows the results of a bivariate analysis of the proportions of school truancy among adolescents in Sierra Leone based on various variables. Older adolescents (≥ 15 years) are more likely to be truant than younger adolescents (≤ 14 years). 48.8% of the girls and 51.2% of the boys engaged in truancy. Adolescents who have attempted suicide (25.6%) are more likely to be truant than those who have not attempted suicide. Adolescents who use alcohol (22.1%) are more likely to be truant than those who do not use alcohol. Adolescents who have ever had sexual intercourse (41.3%) are more likely to be truant than those who have never had sexual intercourse. Adolescents who have ever used marijuana (8.4%) are more likely to be truant than those who have never used marijuana. Adolescents who use amphetamines (13.8%) are more likely to be truant than those who do not use amphetamines. Adolescents who have low parental monitoring (42.6%) are more likely to be truant than those who have high parental monitoring. Sex, suicidal ideation, planned suicide, anxiety, feeling lonely, close friends, bullying, peer support and parental understanding were not associated with truancy among adolescents in Sierra Leone.

**Table 1:** Bivariate analysis of the proportions of school truancy among adolescents in Sierra Leone (*n* = 2769)

Variables	School Truancy		
	No *n* (%)	Yes *n*(%)	*p*-value
**Age (years)**			0.012
≤ 14 years	661(38.2)	300(30.5)	
≥ 15 years	1103(61.8)	688(69.5)	
**Grade**			0.062
Junior secondary school (JSS)	862(38.9)	403(31.0)	
Senior Secondary School (SSS)	896(61.1)	585(69.0)	
**Sex**			0.907
Female	956(48.5)	514(48.8)	
Male	793(51.5)	450(51.2)	
**Anxiety**			0.115
No	1470(82.8)	773(79.0)	
Yes	300(17.2)	213(21.0)	
**Suicidal ideation**			0.855
No	1486(86.2)	797(85.6)	
Yes	242(13.8)	144(14.4)	
**Planned suicide**			0.773
No	1476(84.5)	786(83.5)	
Yes	277(15.5)	168(16.5)	
**Attempted suicide**			0.003
No	1482(84.8)	721(74.4)	
Yes	272(15.2)	258(25.6)	
**Felt lonely**			0.215
No	1432(81.9)	751(78.4)	
Yes	331(18.1)	227(21.6)	
**Close friends**			0.370
No	160(9.7)	86(8.4)	
Yes	1584(90.3)	876(91.6)	
**Bullied**			0.101
No	1349(84.1)	700(80.2)	
Yes	254(15.9)	172(19.8)	
**Alcohol use**			< 0.001
No	1531(91.4)	677(77.9)	
Yes	158(8.6)	227(22.1)	
**Ever had sexual intercourse**			< 0.001
No	1287(76.5)	549(58.7)	
Yes	411(23.5)	371(41.3)	
**Peer support**			0.415
No	1208(69.8)	685(71.5)	
Yes	555(30.2)	292(28.5)	
**Ever used marijuana**			< 0.001
No	1685(98.0)	839(91.6)	
Yes	33(2.0)	100(8.4)	
**Amphetamine use**			0.001
No	1298(95.2)	586(86.2)	
Yes	66(4.8)	119(13.8)	
**Parental monitoring**			< 0.001
No	853(46.9)	581(57.4)	
Yes	895(53.1)	397(42.6)	
**Parental understanding**			0.108
No	942(53.0)	592(58.7)	
Yes	818(47.0)	388(41.3)	
**Parental bonding**			0.074
No	911(51.4)	609(58.5)	
Yes	840(48.6)	375(41.5)	
**Parental intrusion of privacy**			0.960
No	1230(70.6)	686(70.7)	
Yes	529(29.4)	290(29.3)	

*p*-values from Chi-square test

### Prevalence of school truancy among adolescents in Sierra Leone

**Fig. 1:**
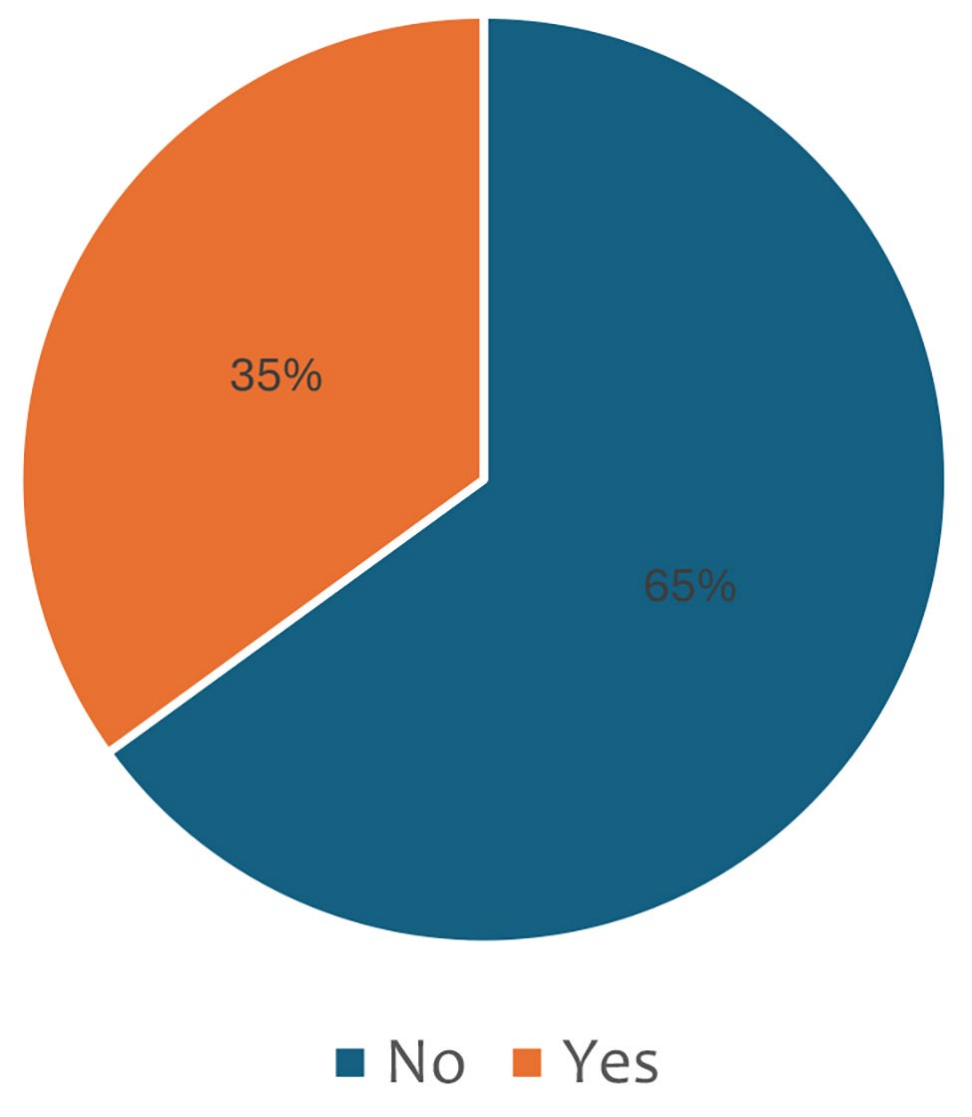
Prevalence of school truancy among adolescents in Sierra Leone

Figure 1 shows the prevalence of school truancy among adolescents. The results indicated that few (35%) of Sierra Leonean adolescents were truants in school.

### Predictors of school truancy among adolescents in Sierra Leone

Table 2 shows the predictors of school truancy among adolescents in Sierra Leone. Adolescents who use alcohol (AOR = 2.28, 95% CI = 1.45, 3.58) and who have ever had sexual intercourse (AOR = 1.67, 95% CI = 1.10, 2.53) had higher odds of being associated with school truancy. Adolescents who planned suicide (AOR = 0.58, 95% CI = 0.36, 0.93) and parents did not intrude on their privacy (AOR = 0.66, 95% CI = 0.45, 0.97) had lower odds of being associated with school truancy.

**Table 2:** Predictors associated with school truancy among adolescents in Sierra Leone

	School truancy
Variables	aOR [95% CI]
**Age (years)**	
≤ 14 years	1.00
≥ 15 years	1.19 [0.83, 1.69]
**Grade**	
JSS	1.00
SSS	1.18 [0.80, 1.73]
**Sex**	
Female	1.00
Male	0.76 [0.56, 1.04]
**Anxiety**	
No	1.00
Yes	1.44 [0.93, 2.26]
**Suicidal ideation**	
No	1.00
Yes	0.62 [0.26, 1.38]
**Planned suicide**	
No	1.00
Yes	0.58** [0.36, 0.93]
**Attempted suicide**	
No	1.00
Yes	1.45 [0.79, 2.67]
**Felt lonely**	
No	1.00
Yes	1.04 [0.57, 1.91]
**Close friends**	
No	1.00
Yes	1.23 [0.52, 2.88]
**Bullied**	
No	1.00
Yes	1.06 [0.71, 1.58]
**Alcohol use**	
No	1.00
Yes	2.28*** [1.45, 3.58]
**Ever had sexual intercourse**	
No	1.00
Yes	1.67** [1.10, 2.53]
**Peer support**	
No	1.00
Yes	1.03 [0.75, 1.43]
**Ever used marijuana**	
No	1.00
Yes	2.73 [0.73, 10.24]
**Amphetamine use**	
No	1.00
Yes	1.54 [0.72, 3.28]
**Parental monitoring**	
No	1.38 [0.98, 1.96]
Yes	1.00
**Parental understanding**	
No	1.17 [0.78, 1.77]
Yes	1.00
**Parental bonding**	
No	0.97 [0.73, 1.29]
Yes	1.00
**Parental intrusion of privacy**	
No	0.66** [0.45, 0.97]-
Yes	1.00

aOR = adjusted odds ratios; CI confidence interval; ** p<; 0.01, *** p<; 0.001

## Discussion

This study examined the prevalence and predictors of school truancy among adolescents in Sierra Leone. Our study found a prevalence rate of 35% of school truancy among adolescents in Sierra Leone. The possible reasons for the high prevalence of school truancy among adolescents in Sierra Leone may be attributed to dilapidated schools, underqualified teachers, and large class sizes, which can make learning demotivating and unproductive [10]. This can be addressed by conducting comprehensive assessments of school buildings to identify repairs and renovations needed—Prioritise schools in the worst conditions that pose safety hazards. Provide ongoing professional development programs for teachers to enhance their skills and knowledge in pedagogy, subject areas, and classroom management. Equip schools with essential technology tools like computers, projectors, and internet access to enhance learning experiences. Violence and unsafe learning environments can create anxiety and fear, causing students to avoid school [7]. The curriculum may not be engaging or relevant to the student’s lives and aspirations, leading to disinterest and truancy. Undiagnosed learning disabilities or lack of support for students with special needs can lead to frustration and truancy. Depression, anxiety, or other mental health challenges can significantly impact attendance and engagement in school [13]. Some students may not see the value of education or lack clear goals for the future, leading to poor attendance. Therefore, to address truancy among adolescents in schools, the government and partner organisations in Sierra Leone should ensure free and accessible education for all, including addressing hidden costs and improving infrastructure in rural areas. Improve school quality through teacher training, updated curriculum, and addressing violence and bullying. Provide mental health support and learning assistance for students facing challenges. By addressing these underlying issues and implementing holistic solutions, Sierra Leone can work towards reducing school truancy and ensuring quality education for all its adolescents.

Our study found that adolescents who used alcohol had higher odds of being associated with school truancy. Our findings are consistent with previous studies in Ghana [5], Zambia [2], Southeast Asia nations [15], and in Malaysia [6]. Alcohol use can impair judgment and increase impulsivity, making it more likely for adolescents to make decisions like skipping school. Hangover symptoms like tiredness, headaches, and nausea can make it difficult for students to attend school or concentrate in class [19]. Alcohol use can lead to a lack of motivation and decreased interest in schoolwork, contributing to truancy [20]. A study on the specific dynamics between alcohol use and truancy in Sierra Leone is needed. Such research could inform targeted interventions and support programs simultaneously addressing both issues.

Our study found that adolescents who have had sexual intercourse had higher odds of being associated with school truancy in Sierra Leone. Our study is consistent with the previous study [13]. As adolescents experience sexual feelings and relationships, their focus might shift away from academics and towards their new social experiences. This can lead to decreased interest in school and skipping classes. Early sexual activity can be met with stigma and shame in some Sierra Leonean communities [21]. This can cause adolescents to avoid school, fearing judgment or bullying from peers or teachers. Adolescents who engage in early sexual activity might be more prone to other risky behaviours, including skipping school [22]. Early sexual activity can lead to unplanned pregnancies and childcare needs, placing financial strain on families [20]. This can force adolescents to work or contribute to household responsibilities, impacting their ability to attend school. The government should ensure that schools avoid victim-blaming or stigmatising adolescents who engage in early sexual activity. They should also focus on providing comprehensive sexuality education that empowers adolescents to make informed decisions about their sexual health and relationships. By taking these steps, we can create a supportive environment where all adolescents in Sierra Leone can thrive, regardless of their sexual choices.

Both alcohol use and sexual activity can negatively impact academic performance [23]. Hangovers, fatigue, and emotional distress can make it difficult to focus on school and can further disrupt attendance [24]. Students who are part of social groups that normalize alcohol use or risky sexual behavior are more likely to engage in increased absenteeism [22, 25].

Our study found that adolescents who have planned suicide had lower odds of being associated with school truancy in Sierra Leone. The finding that adolescents who planned suicide attending school more often than those who did not seems counterintuitive, as it contradicts the common assumption that suicidal individuals are likely to disengage from various aspects of life, including school. Perhaps pre-existing social isolation contributed to suicidal thoughts, and school might be the only remaining social outlet. Alternatively, the planned suicide itself might have led to isolation, and attending school could be an attempt to reconnect. Adolescents who planned suicide may have been more attached to school as a source of hope or support and thus less likely to skip school [26]. Studies on the specific factors that influence suicidal behaviours and school attendance among this population are needed in Sierra Leone.

The findings from our study revealed that adolescents whose parents did not intrude on their privacy had lower odds of being associated with school truancy in Sierra Leone. Adolescents whose parents do not intrude on their privacy may have more trust and communication with their parents and, thus, are more likely to follow their guidance and expectations regarding school attendance [27]. Adolescents whose parents do not intrude on their privacy may have more autonomy and responsibility for their decisions and actions, thus more motivated to attend school and achieve their goals. Adolescents whose parents do not intrude on their privacy may have more positive self-esteem and self-efficacy and, therefore, less likely to be influenced by negative peer pressure or social norms to skip school [28]. It is important to note that constant monitoring or snooping can damage trust between parents and adolescents [29]. This can lead to adolescents withholding information about their activities, including skipping school, to avoid confrontation. Constant monitoring can create a sense of anxiety and a lack of control over their lives in adolescents. This anxiety can make them avoid school as a coping mechanism [30]. Programs should educate parents on respecting age-appropriate autonomy as their children mature. This allows adolescents to develop a sense of responsibility and problem-solving skills. Interventions should promote open and honest communication between parents and adolescents. This can involve workshops or programs teaching communication skills and respectful dialogue strategies.

### Policy and practice implications

The study highlights a concerningly high prevalence of school truancy among adolescents in Sierra Leone. This suggests a critical need for interventions to address this issue and promote educational engagement. Government and partner organisations should develop and implement comprehensive truancy prevention programs within schools, addressing underlying causes like academic difficulties, peer pressure, and lack of motivation. They should Invest in mental health interventions and support services for adolescents, particularly those with suicidal ideation, to address underlying emotional struggles that could contribute to truancy. They should implement programs and resources to improve parent-child communication and promote healthy boundaries, empowering adolescents while providing appropriate guidance and support. They should partner with community organisations and leaders to create safe and supportive environments for adolescents, offering alternative activities and addressing potential risk factors like substance abuse and early sexual engagement.

### Strengths and limitations

One of the vital strengths of the study is that the GSHS provides data from a large, randomly selected sample of adolescents across Sierra Leone, ensuring the generalizability of findings to the entire adolescent population. Using consistent questionnaires and data collection protocols across countries allows for reliable comparisons and avoids potential biases in individual studies. The GSHS includes a wide range of variables related to individual characteristics, health behaviours, family environment, and school factors, allowing for the exploration of multiple potential predictors of truancy. This study, however, has some limitations. The cross-sectional research makes establishing causal relationships between factors and truancy difficult. Reliance on self-reported information about sensitive topics like alcohol use and sexual activity may introduce bias due to underreporting or social desirability. The GSHS does not directly measure truancy but uses proxy indicators like skipping school in the past month. This may not capture all forms of truancy, potentially underestimating the true prevalence. The GSHS focuses on in-school adolescents, potentially missing those who have already dropped out due to truancy, limiting the complete picture of the issue.

## Conclusion

An alarmingly high prevalence of school truancy was identified among adolescents in Sierra Leone. This underscores the urgency of addressing the issue and implementing impactful interventions to promote educational engagement. Alcohol use and ever having sexual intercourse were significantly associated with increased odds of truancy, highlighting the potential influence of risky behaviours on school attendance. This suggests a need for interventions to raise awareness about these risks and support adolescents in making healthy choices while remaining in school. Interestingly, the study found that adolescents who planned suicide and those with parents who intruded on their privacy had lower odds of truancy. This requires further investigation into the underlying reasons and potential mediating factors. It suggests that interventions must be nuanced and consider the complex interplay of mental health, family dynamics, and school attendance.

## Data Availability

The dataset for this analysis was obtained from the GSHS–Sierra Leone, 2017. Access to the data can be obtained at the WHO website: https://extranet.who. int/ncdsmicrodata/index.php/catalog/879.
